# Redundant in the light, indifferent to the heat: the role of the PIF family in mature tomato

**DOI:** 10.1093/plphys/kiag380

**Published:** 2026-06-16

**Authors:** Hannah Rae Thomas

**Affiliations:** Assistant Features Editor, Plant Physiology, American Society of Plant Biologists; Department of Horticulture, Zijingang Campus, Zhejiang University, 866 Yuhangtang Road, Hangzhou PR China

Plants have evolved complex mechanisms to meet their need for sunlight, known as the shade avoidance syndrome (SAS). SAS includes stem elongation, leaf hyponasty (abaxial growth), and thin, pale leaves, among other traits, when plants are grown under low light (Smith and Whitelam 1997). Plants perceive shade by measuring the red:far-red (R:FR) light ratio via PHYTOCHROME (Phy) A and B photosensors (Ballaré et al. 1990). Shade has a low R:FR, which inactivates PhyB (Casal 2013). PhyB also senses temperature in addition to light (Legris et al. 2016). At high temperatures, PhyB is inactivated, which releases its inhibitory effect on various transcription factors, including PHYTOCHROME-INTERACTING FACTORS (PIFs) (Park et al. 2004). PIFs are central regulators of SAS and thermomorphogenesis in *Arabidopsis thaliana (*Legris et al. 2016*)*. PIFs in Arabidopsis promote the expression of genes involved in auxin biosynthesis and growth, leading to taller seedlings in the shade and at elevated temperatures (Hornitschek et al. 2012; Cañibano et al. 2025).

Much of what we know about light and temperature sensing comes from studies in Arabidopsis, a cold-tolerant plant. In contrast, tomato (*Solanum lycopersicum)* evolved in a warm climate. So, while there are similarities in the function of PIFs in Arabidopsis and tomato, it is logical that they exhibit divergent physiological responses to high temperature. For example, hypocotyl elongation in Arabidopsis is largely controlled by auxin alone, whereas tomato requires auxin, brassinosteroids, and gibberellins. Furthermore, AtPIF7 is the primary driver of SAS-induced hypocotyl elongation in Arabidopsis (Li et al. 2012), whereas previous work by Kunta, et al. (2025) identified SlPIF8a as the key driver of SAS-induced growth, with partially redundant activity from SlPIF4/7a/7b. Interestingly, in the same study, SlPIF4, 7a, 7b, or 8a were found to be independent of heat-induced hypocotyl elongation, but it was unclear whether other SlPIFs might be responsible.

Despite significant progress in understanding the roles of light and temperature regulation in plant growth, substantial functional gaps remain in how PIFs regulate growth in crops, especially under field conditions that lack well-controlled environmental conditions. Additionally, it is unclear how redundant the 8 tomato PIFs are.

Recently, in *Plant Physiology*, Kunta et al. (2026) set out to characterize the role of the entire *SlPIF* family in regulating tomato growth. To do this, they generated an octuple PIF mutant, termed *slpifo*, in addition to a quadruple-mutant (*slpifq*) lacking functional *PIF4/7a/7b/8a*, and explored the responses of 21-day-old plants to multiple growth and stress conditions. The authors first showed that the wild-type (WT) tomato exhibited significantly elongated stems at both high temperatures (30 °C) and under far-red light, but that only the FR-induced growth was perturbed in *slpifq* and more so by *slpifo* (Fig. 1a–1b). This surprising finding contradicts previous reports that tomato, like Arabidopsis, exhibits heat-induced elongation due to PIF expression during the seedling stage (Rosado et al. 2019; Zhu et al. 2024). To validate their work in mature plants, they also subjected *slpifq* and *slpifo* seedlings to high temperature and found that the *slpif1a/1b/3* and *slpifq* mutants showed heat-induced growth similar to WT. In contrast, only the *slpifo* mutants showed reduced, but not completely absent, heat-induced growth. From this, the authors concluded that tomato only displays PIF-dependent thermomorphogenesis at the seedling stage, whereas in 21-day-old tomato, heat-induced growth is independent of PIFs.

**Figure 1:**
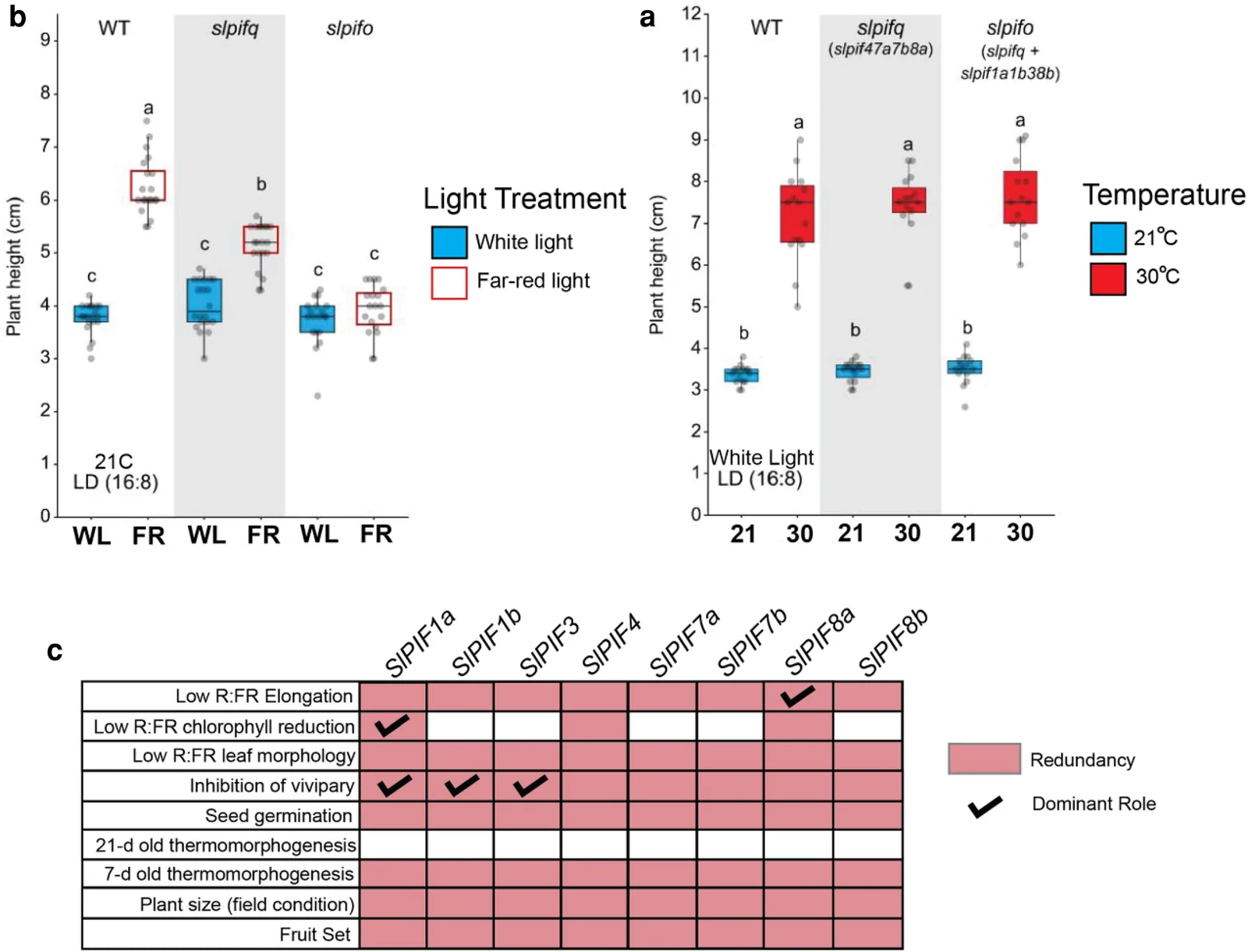
SlPIFs are redundant or partially redundant for developmental processes, including shade-avoidance syndrome, but are not involved in thermomorphogenesis in 21-day-old tomato. (a) 21-day-old WT, *slpifq*, and *slpifo* tomato plants were grown at 21 °C under long-day white light (WL) or far-red light (FR). FR increased plant height as part of shade avoidance in WT. The *slpifo* plants lacked FR-induced growth, whereas *slpifq* showed an intermediate phenotype. This supports previous reports that SlPIFs are key for FR-induced growth, with functional redundancy within the gene family. (b) 21-day-old wild-type (WT), *slpifq* (*slpif4/7a/7b/8a*), and *slpifo* (*slpif1a/1b/3/4/7a/7b/8a/8b*) tomato plants were grown under long-day conditions (16:8) with white light at either 21 °C or 30 °C. The *slpifq* and *slpifo* mutations did not affect the thermomorphogenesis phenotype, indicating that heat-induced elongation is independent of SlPIFs at this stage. A, B) Adapted from Kunta et al. 2026. (c) The redundancy of *SlPIFs* was further elucidated. FR-induced stem elongation, FR-induced alterations in leaf morphology and inhibition of vivipary, seed germination, plant size, and fruit set were PIF-dependent and exhibited broad functional redundancy. In contrast, chlorophyll reduction under low F:FR was due to PIF1a, PIF4, and PIF8a, with PIF1a demonstrating a dominant role (black check). The 7-day-old seedlings showed PIF-dependent thermomorphogenesis, whereas no PIFs regulated thermomorphogenesis in 21-day-old plants. Dominant roles for PIF1a/1b/3 were identified in inhibiting vivipary, while the central role of PIF8a during FR-induced growth was previously reported (Kunta et al. 2025).

The authors next explored how SlPIFs regulate multiple growth phenotypes. They first demonstrated that low R:FR light leads to larger leaves in a PIF-dependent manner in WT. FR typically produces larger, thinner leaves in WT, but *slpifo* lacked such light-response changes. Furthermore, even in white light, *slpifo* showed smaller, thinner leaves than WT, suggesting that in tomato, PIFs also have functions in plant development that are independent of SAS.

Next, the authors investigated the role of SlPIFs in shade-induced leaf paleness. They first showed that, when subjected to far-red light, WT and *slpifq* formed leaves with less chlorophyll than those of *slpifo*. This indicated that although *slpifq* lacks 4 PIFs, the remaining 4 provide enough functional redundancy to maintain the WT phenotype. To identify the minimum number of PIFs that regulate this process, the authors conducted a well-designed classical genetics experiment by crossing the pale *slpifq* mutant with the dark green *slpifo* mutant and analyzing the segregation pattern in the F2 generation. They identified 17 dark green plants, all of which contained a mutation in *PIF1a*. This indicated that in the *pifq* background, where *PIF4*, *7a*, *7b*, and *8a* are already altered, loss of *PIF1a* is sufficient to perturb the pale phenotype. Next, to determine whether PIF1a alone is sufficient to regulate this phenotype or whether other PIFs show redundant function, they backcrossed *slpif1a/q* (*slpif1a*, *4*, *7a*, *7b*, *8a*) against WT. They determined the segregation pattern of the F2 generation. If only one gene controlled the chlorophyll phenotype, the F2 generation would segregate 1:4 (pale:dark green); if 2 genes, 1:16; and if 3 genes, 1:64. The authors found that 2:128 (roughly 1:64) were dark green, indicating that 3 PIFs are involved. To validate which other PIFs are needed in addition to PIF1a, they crossed *slpif1a/q* × *slpif8a*. This led to a segregation pattern close to 1/16. Since in this cross, *pif8a* is homozygous for the mutation, only *PIF1a* and one additional gene are segregating. To validate the last gene, they crossed *slpif1a/q* × *slpif4* and identified the same segregation pattern. This confirmed that SlPIF1a, SlPIF4, and SlPIF8a are functionally redundant in the SAS chlorophyll phenotype, while SlPIF1a plays a major role.

Lastly, the authors showed that, in addition to growth, SlPIFs are also involved in fruit and seed development. When grown in open fields and greenhouses, *slpifo* showed slower growth, fewer and smaller fruits, and delayed germination compared to WT. They also found that a significant number of *slpifo* fruits already contained germinated seeds, a process called vivipary. They further narrowed down which SlPIFs are involved in this process by showing that *slpif1a/1b/3* retained the vivipary phenotype, but to a lesser extent than the octuple mutant.

Overall, the authors found that while SlPIFs are essential for SAS in tomato, and that thermomorphogenesis is largely independent of PIFs in older tomato plants, especially those grown under field conditions. They also found that SlPIFs are involved in a broad range of developmental processes, including leaf and seed development, independent of light signaling, suggesting that the role of PIFs has expanded in tomato.

Despite a significant advance in our understanding of the PIF family in tomato, several questions remain unanswered. For example, in contrast with this work, previous papers have shown that growth in tomato seedlings is PIF-dependent (Rosado et al. 2019; Zhu et al. 2024). It is unclear whether these regulatory pathways are active only in seedlings or whether alternative mechanisms act in mature tissues. The authors also discuss this point and posit that other known regulators of thermomorphogenesis, such as HY5 or COP1, might have novel or conserved roles in mature tomato as well (Lee et al. 2021). The authors also showed that in tomato, leaf morphology and seed germination are regulated by PIFs independently of changes in light or temperature. This raises interesting questions about the mechanism by which PIFs act in these pathways, how their expression is induced, and which target genes they act on.

Modulating PIFs is often cited as a potential approach to improve crop production, but Kunta et al. (2026) show that converting a shade-sensitive plant to a shade-insensitive plant also comes with negative tradeoffs, including reduced growth, fewer fruits, and poor seed quality. The authors suggest that to positively modulate the shade response to benefit production, parallel mutations in growth and reproduction are also required. Indeed, a more elegant approach might target the deleterious responses of SAS rather than PIFs alone. And most importantly, it remains imperative that mechanisms such as SAS be tested in crop systems under experimental variables that most closely reflect production conditions.

**Recent related articles in *Plant Physiology*:**

- Sangwon Cho, Giltsu Choi. 2025. Phytochrome B regulates cortical microtubule arrangement to control cotyledon polar expansion by repressing *LONGIFOLIA*s. *Plant Physiology*. 198 (1):kiaf162. https://doi.org/10.1093/plphys/kiaf162
- Fichman Y, et al. 2024. Rapid plant-to-plant systemic signaling via a *Cuscuta* bridge. *Plant Physiology*. 196(2):716–721. https://doi.org/10.1093/plphys/kiae339
- Lee JH, Doan TM, Bruzual A, Senthilkumar S, Yoo CY. Light-regulated dual-targeting of NUCLEAR CONTROL OF PEP ACTIVITY establishes photomorphogenesis via interorganellar communication. 2025. *Plant Physiology*. 199(1):kiaf289. https://doi.org/10.1093/plphys/kiaf289

## Data Availability

Not applicable.
